# Psychodynamic Psychotherapy for Postpartum Depression: A Systematic Review

**DOI:** 10.1007/s10995-023-03655-y

**Published:** 2023-04-08

**Authors:** N. Valverde, E. Mollejo, L. Legarra, M. Gómez-Gutiérrez

**Affiliations:** 1 Faculty of Psychology, Department of Assessment, Personality and Clinical Psychology, Complutense University of Madrid, Campus of Somosaguas s/n, Madrid, 28040 Spain; 2 Department of Psychiatry and Mental Health, Southeast University Hospital of Madrid, Madrid, Spain

**Keywords:** Women’s mental health, Perinatal period, Postpartum depression, Psychodynamic therapy, Systematic review

## Abstract

**Objectives:**

Postpartum depression estimated prevalence in women is between 5 and 26% and it has adverse effects both on the mother, infant and her partner. Psychological treatments have proved to be effective for women with mild-to-moderate symptoms. Whereas several systematic reviews have assessed the effects of different psychological interventions for postpartum depression, such as cognitive-behavioural therapy or interpersonal therapy, no review assessing psychodynamic therapy has been carried out. A systematic review was conducted to evaluate the efficacy of psychodynamic therapy for postpartum depression.

**Methods:**

Studies were identified using the following databases: PsycINFO, Psycarticles and Pubmed over January 2023. The requirements for the studies were the following: they had to be quantitative, available in English, including a psychodynamic intervention targeting treatment or prevention of postpartum depression which starts during pregnancy or within the first 12 months after giving birth. Case studies, qualitative studies or studies focused on improving parent-infant relationship or infant outcome were excluded from this research.

**Results:**

Seven trials including 521 women met the inclusion criteria. In summary, three randomized controlled trials and four longitudinal studies were found. The most frequently used assessment tool was EPDS, five were individual interventions and the other two were group interventions.

**Discussion:**

All studies reported the efficacy of psychodynamic interventions for postpartum depression, both in home and clinical settings and both in group and individual format. The limited number of trials, small sample sizes and lack of appropriate control groups were the main limitations.

**Conclusions for practice:**

Psychodynamic therapy is probably efficient intervention for postpartum depression. Future research with strong methodological designs is needed to confirm these findings.

**Significance:**

What is already known on this subject? Several systematic reviews have assessed the effects of different psychological interventions for postpartum depression, but no review assessing psychodynamic therapy has been carried out. What this study adds? A systematic review was conducted to evaluate the efficacy of psychodynamic therapy for postpartumdepression. This makes the systematic review a unique contribution to the literature.

## Introduction

Historically parent’s mental health during the perinatal period has been overlooked, as it was conceived as a time of joy and emotional stability. Only in 1950’s some authors started to write about maternity blues, postpartum depression, and puerperal psychosis (Besser et al., 2008). Studies have generally targeted mothers and their psychological wellbeing. On the contrary, paternal mental health remains under-investigated even though non-gestational parents may also suffer from mental disorders in the peripartum period.

Postpartum depression encompasses a depression disorder occurring within the 12 months following childbirth (Branquinho et al., 2021) and the disorder can be compared to a major depressive episode in any moment of a woman’s life. However, symptoms such as anxiety, anhedonia, aggressive obsessional thoughts, restlessness or concentration and decision-making difficulties are more frequent or severe in the immediate postpartum (Batt et al., 2020). Postpartum depression has adverse effects on the woman and, therefore, on the infant as the disorder is closely related to difficulty to carry out different parenting tasks, such as breastfeeding, sleeping, or responding to the infant’s needs (Branquinho et al., 2021; Nanzer et al., 2012). It is also the strongest predictor of paternal depression during the perinatal period (Kaźmierczak et al., 2020). In addition, it is one of the main causes of maternal deaths in the first year after childbirth as the mother may develop suicidal thoughts and intentions. (Al-Halabí et al., 2021). The estimated prevalence of postpartum depression in women varies across countries, assessment criteria or time frame ranging from 5 to 26% (Liu et al., 2022).

Postpartum depression is still significantly unrecognised and undertreated, as only between 13 and 18% of women, who meet criteria for major depressive disorder, seek treatment during pregnancy and postpartum, due to the deficient knowledge about postpartum psychological disorders, mental health stigma and lack of time when nurturing (Nillni et al., 2018). If not treated, postpartum depression can last three to six months, and 30% of these women will still be depressed one year after giving birth (Nanzer et al., 2012). Fortunately, awareness about women’s mental health is growing progressively, and institutions worldwide recommend screening through the perinatal period.

Psychological interventions such as cognitive-behavioural therapy, interpersonal therapy and psychodynamic therapy have proved to be effective in treating postpartum depression (Cooper et al., 2003; Nillni et al., 2018) and considered the first option for women with mild-to-moderate symptoms. Other interventions, such as psychosocial or supportive interventions, psychoeducation or physical activity, present weak evidence (Werner et al. 2015). Pharmacological treatments have not proved to be superior to either psychological or combined treatments (De Crescenzo et al., 2014).

Several systematic reviews have assessed the efficacy of different interventions for postpartum depression. However, to our knowledge, no systematic review has been carried out to assess psychodynamic therapy. There is scarce evidence for the perinatal period even though psychodynamic therapy has proved to be effective for depression in general population.

The aim of the present systematic review is (1) to synthesise the evidence on the efficacy of psychodynamic psychotherapy for postpartum depression in women; (2) compare it with other psychological interventions and control conditions; and (3) to examine if these results are maintained in the long-term.

This manuscript has not been based on clinical studies or patient data.

## Method

### Search Procedure

This systematic review was conducted according to PRISMA Guidelines (Moher et al., 2009) during the whole month of January 2023 with no date-of-publishing criteria being applied. Studies were identified through the following databases: PsycINFO, Psycarticles and Pubmed. The search terms combined were: Postpartum Depression OR Perinatal Depression OR Postnatal Depression AND Psychodynamic therapy OR Psychodynamic Psychotherapy OR Psychoanalytic Therapy OR Psychoanalytic Psychotherapy. Bibliographic search was completed by reviewing the studies identified below.

### Selection Criteria

**Fig. 1:**
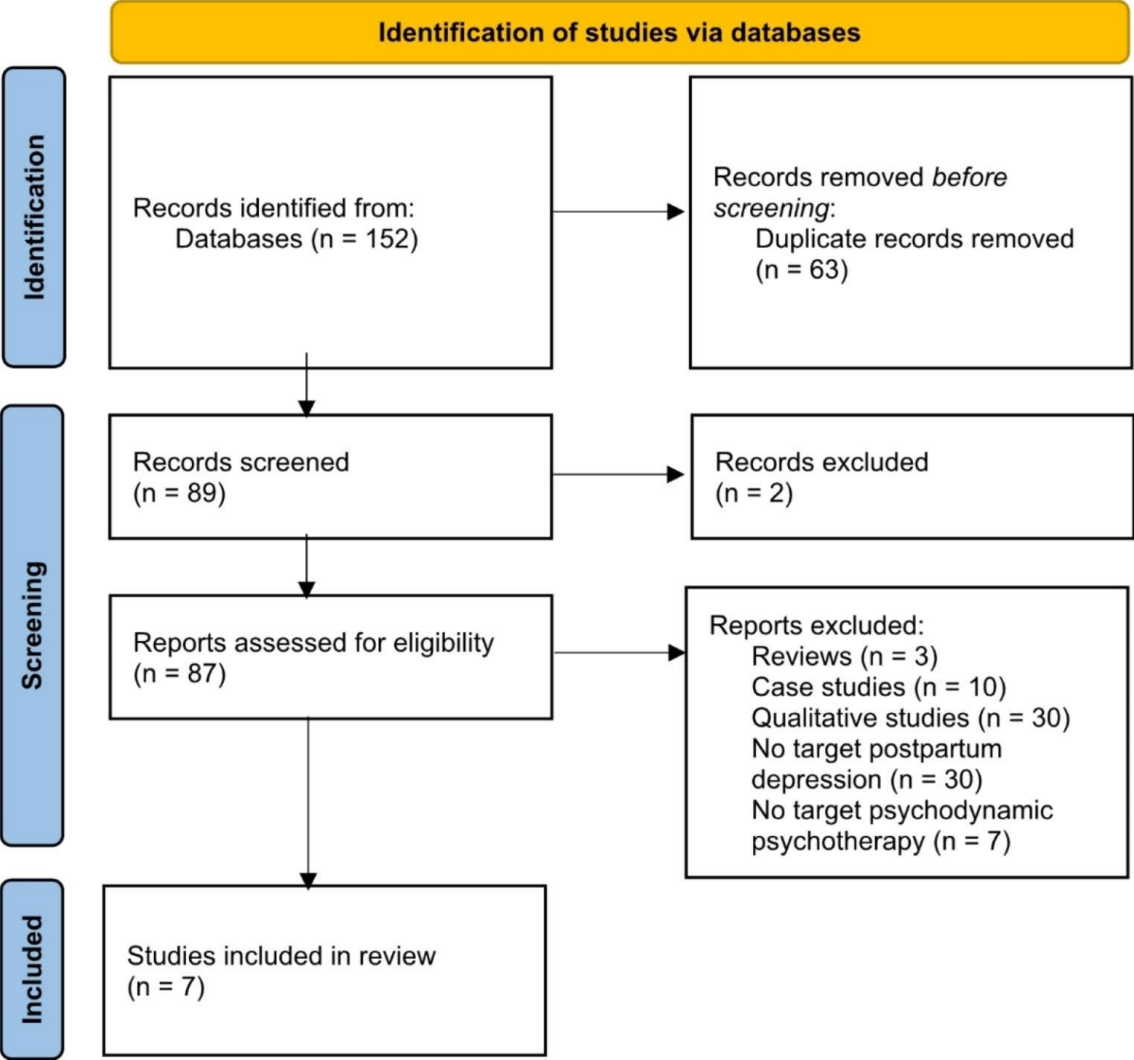
PRISMA flow chart illustrating the identification of included studies

Studies had to meet the following inclusion criteria in order to be included in the review: (1) to be available in English language, (2) to be carried out as quantitative studies, (3) to include a psychodynamic or psychoanalytic intervention, (4) to include an intervention that targets treatment or prevention of postpartum depression, (5) the intervention had to start during pregnancy or within the first 12 months after birth, (6) and finally, to provide outcome measure. Studies were excluded from this research when they met one of the following exclusion criteria: (1) case studies, (2) qualitative studies, (3) studies focused on improving mother-infant or parent-infant relationship or infant outcome. The PRISMA flow diagram in Fig. 1 below presents the number of records considered at each stage of the review.

## Results

The search strategy yielded a total of 87 papers after removing duplicates. Articles were assessed for eligibility and seven papers met inclusion criteria after reviewing the titles and abstracts. Table 1 synthesises characteristics of the seven included studies and offers information about the following data: authors and year of publication; trial type and design; sample size; drop-out rate; measure instruments; intervention approach (treatment or prevention); full length, number of sessions, follow-up and main results.

**Table 1:** Study characteristics

Study	Type design/ Variables	Trial type/ Prevention or treatment	Participants/ Dropout rate	Instruments	Intervention	Duration/ Nº of sessions/ Follow up	Results
Bloch et al., 2012	RCT Postpartum Depression	Treatment PPD	NTOTAL = 42 IG = 20 CG = 22 5%	MADRS SCID EPDS MHI CGI-S CGI-I UKU Side Effect Rating Scale	IG: brief dynamic psychotherapy + sertraline CG: brief dynamic psychotherapy + placebo pills	12 weeks 12 sessions 8 weeks sertraline or placebo Pre, weeks 0, 2, 4, 6, 8 and 12	Significant time effect for depression scores in both groups (MADRS and EPDS) at 8 weeks No group-time interaction effect Overall response rate 62,5% Total remission rate 67,5% at 8 weeks, (82% in IG and 94% in CG at 12 weeks No significant difference between groups
Clarici et al., 2015	RCT Personality traits: narcissistic and depressive Postpartum depression	Treatment PPD	NTOTAL = 16 IG = 5 CG = 11 ---	Beck list criteria SWAP EPDS HRSD ANPS	IG: brief psychodynamic therapy + Intranasal oxytocin CG: psychodynamic therapy and placebo	12 weeks 12 sessions 15 weeks of daily dose of oxytocine or placebo Pre, post week 15	No significant differences in depressive symptoms between IG and CG Both groups significant decrease in HRSD IG: decrease in narcissistic trait CG: decrease depressive trait
Cooper et al., 2003	RCT Postpartum depression Mother-infant relationship Infant behaviour Infant cognitive development Infant attachment	Treatment PPD Postpartum period	NTOTAL = 193 IG1 = 48 IG2 = 43 IG3 = 53 CG = 52 10%	EPDS SCID Therapist rating scale	IG1: non directive counselling IG2: cognitive-behavioural therapy IG3: psychodynamic therapy GC: treatment as usual	12 weeks 8 to 18 weeks postpartum Pre, post, 9 and 18 months postpartum, 5 years postpartum	All treatment groups experienced benefit after treatment Control group did not change in terms of mood during the 10 weeks Only psychodynamic therapy produced a rate of reduction in depression (SCID) Benefits were no longer apparent at 9 months postpartum Treatment did not reduce subsequent episodes of PPD
Kurzweil, 2008	Global functioning	Treatment PPD Postpartum period	N TOTAL = 49 IG = 49 37%	Clinical interview Ad hoc questionnaire GAF	IG: analytic group therapy	Mean: 8 months postpartum Bimonthly sessions Pre, post treatment	Mean GAF increased significantly from pre: 57.5 to post: 66.11 (large effect size) Patients were self-referred No control group
Kurzweil, 2012	Global functioning	Treatment PPD Postpartum period	N TOTAL = 58 IG1 = 23 IG2 = 20 IG3 = 15 33%	Clinical interview Ad hoc questionnaire GAF	IG1: individual psychodynamic therapy IG2: analytic group therapy IG3: individual + group psychodynamic therapy	Mean: 17 month postpartum Pre, post treatment	All groups improved significantly in GAF from pre: 58.05 to post: 68.00 Group condition: self-determined Best results in IG1: Individual treatment Patients were self referred No control group
Moayedoddin et al., 2013	Longitudinal study Depressive and anxiety symptoms Parent infant relation	Treatment Postpartum period	GTOTAL = 34 32%	EPDS STAI SCID GAF CGI PIR GAS	IG: PCP	Mean: 7 sessions Pre, post-treatment	Significant decline in the number of major clinical depressions (13 to 3) Reduction in EPDS clinical scores > 12 (from 76–26%) at post-treatment EPDS X = 9 (still risk score) STAI “non anxius” scores increased from 30–83% at post-treatment Good GAF scores increased from 26–81% at post-treatment No significant improvement found in mother-infant relationship 1 year follow up: EPDS X = 7 Lack of control group
Nanzer et al., 2012	Longitudinal study Depressive symptoms Parent infant relation	Prevention Pregnancy and postpartum period	GTOTAL = 129 IG = 40 CG = 88 IG:14% CG:41% 10%	EPDS DADP GAF PIRGAS	IG: PCP CG: TAU	2 sessions during pregnancy + 2 sessions in postpartum Pre, 3 and 6 months postpartum	Significant decrease from T1 to T2 in IG in EPDS No women in IG meet cut off scores (12) on EPDS at any follow up No significant differences between both groups from T1 to T3 in EPDS scores EPDS scores in IG slightly continues to decrease after T2 Good scores in PIR GAS in the majority of the women undergoing treatment

ANPS: Affective Neuroscience Personality Scale; CGI-S and CGI-I: Severity of Illness and Improvement Scales; CG: Control Group; CGI: Clinical Global Impression; DADP: Dépistage anténatal de la dépression postnatal; EPDS: Edinburg Postnatal Depression Scale; GAF: Global Assessment Functioning; HRSD: Hamilton Rating Scale for Depression; IG: Intervention Group; MADRS: Montgomery-Asberg Depression Rating Scale; MHI: Mental Health Inventory; MHI: Mental Health Inventory; PIR GAS: Parent-Infant Relationship Global Assessment Scale; PPD: Postpartum Depression; SCID: Structured Clinical Interview for DSM; SWAP: Shedler–Westen Assessment Procedure; UKU: Side Effect Rating Scale

### Design and Trial Type

Three out of all the studies were randomized controlled trials (Bloch et al., 2012; Clarici et al., 2015; Cooper et al., 2003) and four were longitudinal studies (Kurzweil, 2008, 2012; Moayedoddin et al., 2013; Nanzer et al., 2012). A total of five trials focused on the treatment of postpartum depression (Bloch et al., 2012; Clarici et al., 2015; Cooper et al., 2003; Kurzweil, 2008, 2012) whereas one study aimed to prevent postpartum depression which started during pregnancy (Nanzer et al., 2012). The other study considered including both pregnant and puerperal women from the beginning (Moayedoddin et al., 2013).

### Participants

The seven studies included a total of 521 patients. The number of participants ranged from 16 (Clarici et al., 2015) to 193 (Cooper et al., 2003). Only two studies reported samples fewer than 40 (Clarici et al., 2015; Moayedoddin et al., 2013) and two included more than 100 participants (Cooper et al., 2003; Nanzer et al., 2012). Women were recruited during the perinatal period, from the beginning of pregnancy to the period of one year postpartum. Targeted women were pregnant or postpartum women with depressive symptoms. In five of the studies they also met criteria for a major depressive disorder diagnosis, assessed by a structured clinical interview (Bloch et al., 2012; Cooper et al., 2003; Moayedoddin et al., 2013; Kurzweil, 2008, 2012). All the trials excluded severe mental health conditions. The age of women ranged from 17 to 46 years.

Rejection to take part in three of these studies (i.e., percentage of women who were approached but declined to participate) is stated to be 47% in one of them (Moayedoddin et al., 2013), 6% in another (Cooper et al., 2003) and 54% in the third one, which also included those women who were found unsuitable for the protocol (Bloch et al., 2012). Dropout rates range from 5% (Bloch et al., 2012) to 37% (Kurzweil, 2008).

### Instruments

For the assessment of depressive symptoms, the Edinburgh Postnatal Depression Scale (EPDS; Cox et al. 1987) was the most frequently used self-report symptom measurement system, applied in five of the seven trials. The EPDS is a 10-item self-administered questionnaire to rate how participants felt in the previous seven days. It has shown satisfactory sensitivity and specificity and it is a validated tool for screening depressive symptoms in the postpartum.

Other standardized questionnaires used for screening depressive symptomatology were the Hamilton Rating Scale for Depression (HDRS; Hamilton 1967), a multiple-response questionnaire used to provide an indication of depression from the point of view of the evaluator; the Montgomery-Asberg Depression Rating Scale (MADRS; Montgomery and Asberg 1979), whose score is also based on a clinical interview with the patient; the Dépistage Antenatal de la Depression Postnatal, (DADP; Nanzer and Riguetti-Vetelma 2009) a six-item questionnaire that includes four items concerning psychological aspects and two items referring to somatic issues; and ad hoc Beck List Criteria for postpartum depression (Beck, 2001).

Two studies used an author-generated questionnaire to obtain ratings on how helpful the treatment is (Kurzweil, 2008, 2012). The ad hoc questionnaire assessed their sense of interpersonal connection and relationships, general wellbeing, outlook on life and parenting confidence, mood, level of anxiety and frustration tolerance.

Three trials used the Structured Clinical Interview for DSM for the diagnose of Postpartum Depression (First et al., 1994; Shalev et al., 1994; Spitzer et al. 1992) and two trials used a different clinical interview (Kurzweil, 2008, 2012). The Global Assessment Functioning (Jones et al., 1995) a numeric scale included in the DSM, was used in four of the seven studies (Kurzweil, 2008, 2012; Moayedoddin et al., 2013; Nanzer et al., 2012).

### Interventions

Five studies targeted the treatment of postpartum depression (Bloch et al. 2012; Clarici et al. 2015; Cooper et al. 2003; Kurzweil 2008; 2012), one trial focused on both, treatment and prevention with pregnant and postpartum women (Moayedoddin et al., 2013) and one was a preventive study that considered women at risk during pregnancy (Nanzer et al., 2012). Intervention modalities included individual psychotherapy and group therapy and only in Cooper’s trial (2003) sessions were home-based delivered.

Brief dynamic psychotherapy (BDP) in individual format was the most frequently used type of intervention, applied in five out of seven trials. BDP is a time-limited intervention aiming to foster insight regarding repetitive conflicts. Number of sessions ranged from four (Nanzer et al., 2012) to twelve (Clarici et al., 2015). In contrast, Kurzweil’s studies assessed long-term psychodynamic group treatment (2008) and compared it to individual and mixed interventions (2012) in a self- referred study in which women began and abandoned treatment conditions on a self-determined basis. The type of intervention was randomly assigned in three studies (Bloch et al., 2012; Clarici et al. 2015; Cooper et al. 2003). In all the trials, except for one (Clarici et al., 2015) psychodynamic treatments were manualized and in four of them psychotherapists were supervised (Bloch et al., 2012; Clarici et al. 2015; Cooper et al. 2003; Nanzer et al. 2012).

### Main Results of the Interventions

These studies suggest that psychodynamic interventions can be effective for the treatment and prevention of postpartum depression. Adjuvant pharmacological treatment does not seem to improve the results obtained with psychodynamic psychotherapy as Bloch et al. (2012) found that both, brief dynamic psychotherapy (BDP) with placebo and BDP with sertraline, demonstrated to produce significant remission of postpartum depression symptomatology. However, no added benefits were found by adding medication to psychotherapy alone. Similar findings emerged from Clarici’s trial (2015) who found that BDP had a significant effect on ameliorating depressive symptoms but that addition of intranasal oxytocin to BDP did not lead to an improvement in these results.

Cooper’s team (2003) concluded that all three brief psychological treatments (cognitive-behavioural therapy, non-directive counselling and psychodynamic therapy) had a significant impact on improving maternal mood at posttreatment (4.5 months) in contrast to the control group which received treatment as usual. This confirms previous research results asserting that different types of psychological interventions can be equally beneficial for depressed woman in the postpartum (Nillni et al., 2018); even though only psychodynamic therapy was superior in reducing depression diagnoses in the control group.

Kurzweil’s experience with long-term psychodynamic group treatment (2008) found significant improvement in GAF scores and ad hoc questionnaire at post treatment in her clinical setting study, concluding that the individual format had the best results when compared to group or mixed format in 2012.

Geneva trials with a specific psychodynamic therapy for the perinatal period, Psychotherapy centred on parenthood (PCP), led to good results. First, Nanzer’s team noted significant differences on EPDS and GAF scores in the intervention group (pregnant depressive women) compared to the control group (non-depressed). In addition, no women met a clinical score in EPDS after the treatment, compared to 78% at baseline. These results were maintained at follow-up (six months postpartum). Secondly, Moayedoddin’s team (2013) detected that postpartum women consulting an infant-parent clinic who received PCP displayed a significant improvement in almost all milestones of the post-treatment: reduction in major depression diagnoses (SCID), reduction in EPDS scores (yet over the risk cut-off), reduction in anxiety scores and significant increase in GAF scores. There was no control group.

### Methodological Quality Assessment

The methodological quality from the studies was assessed (Table 2) taking into consideration the presence/absence of the following variables (JADAD scale): randomization, appropriate randomization, drop-out and attrition rates, control group and measure of effect size.

**Table 2:** Quality of studies

Author/ Year	Randomized	Properly randomized	Drop-out	Rejections	Control Group	Effect size	Total
Bloch et al., 2012	1	1	1	1	1	1	6
Clarici et al., 2015	1	0	0	0	1	1	3
Cooper et al., 2003	1	1	1	1	1	1	6
Kurzweil, 2008	0	0	1	0	0	1	2
Kurzweil, 2012	0	0	1	0	1	1	3
Moayedoddin et al., 2013	0	0	1	1	0	1	3
Nanzer et al., 2012	0	0	1	1	1	1	4

This methodological analysis highlights not only the small number of trials, but also the lack of high-quality designs concerning psychodynamic psychotherapy and postpartum depression. Only two were properly randomized out of the seven samples (Bloch et al., 2012; Cooper et al., 2003) and from the five that had a control group, only one received treatment (Cooper et al., 2003).

## Discussion

The main outcomes from this systematic review show that a few but promising studies have been conducted underpinning the benefits of psychodynamic psychotherapy for women struggling with depressive symptoms in the postpartum period. The studies were carried out either in a brief individual or in a long-term group format, both in clinical settings and at home. These results reinforce previous research showing that psychodynamic psychotherapy is beneficial for common mental health disorders such as depression and anxiety and, at least, as effective as other psychological treatments (Leichsenring & Klein, 2014; Driessen et al., 2015).

In relation to other types of psychological interventions, BDP has shown to be equally beneficial as cognitive-behavioural therapy and non-directive counselling in one trial that compared BDP to other psychological interventions (Cooper et al., 2003) confirming previous research (Leichsenring & Klein, 2014).

In terms of time-effects only two trials provided data from long-term assessments. Results from Cooper’s study conclude that the benefits of the interventions were no longer apparent at nine or 18 months stage. Furthermore, none of the treatments improved the risk for future postpartum depression episodes when assessed at five years postpartum stage. On the other hand, Nanzer et al. (2012) demonstrated how the improvement in anxiety and depressive symptoms was maintained after treatment and continued to hold at six months follow-up. These results align with conclusions from recent systematic reviews which state that improvements from psychological interventions are maintained in long-term periods from six to twelve months. (Malhi et al., 2021; Branquinho et al., 2021).

Literature and studies for postpartum depression have generally targeted mothers, even though non-gestational parents can also suffer from mental disorders in the peripartum as transition to parenthood is a major life transition for both, women and men. As a result, this transition may increase the vulnerability to psychological disorders. However, to date there has been a little interest in researching mental health in non-gestational parents.

Several systematic reviews have assessed the efficacy of psychological interventions for postpartum depression such as cognitive-behavioural therapy (Li et al., 2022) or interpersonal therapy (Miniati et al., 2014), but no systematic review assessing psychodynamic therapy has been carried out. This makes the systematic review a unique contribution to the literature. Possible barriers of the shortage of psychodynamic interventions trials point towards the difficulty of standardisation, particularly in long-term variants (Malhi et al., 2021).

Future methodological research with large sample sizes, randomized groups, and follow-up data is needed in order to bridge the gap between clinical practice and experimental evidence.

### Limitations

The search was limited to published journal articles in English and a meta-analysis was not conducted. In addition, there were important limitations such as limited number of trials and poor methodological quality in most of them, small sample sizes and lack of appropriate control groups.

## Conclusions

Psychodynamic approach still plays a minor role in the mainstream theory, research, and treatment for postpartum depression, even though the amount of data has kept growing for the last decade. BDP is probably efficient intervention for postpartum depression. Therefore, it is suggested that research should be continued to assess the effectiveness of psychodynamic interventions in postpartum depression compared to other effective treatments.

## Data Availability

Not applicable.

## References

[CR1] Al-Halabí S, García-Haro J, Rodríguez-Muñoz MDLF, Fonseca-Pedrero E (2021). Conducta suicida y periodo perinatal: Entre el tabú y la incomprensión. Papeles del Psicólogo.

[CR2] American Psychiatric Association (1994). *Diagnostic and Statistical Manual of Mental Disorders* (4th ed.).

[CR3] Batt MM, Duffy KA, Novick AM, Metcalf CA, Epperson CN (2020). Is postpartum depression different from depression occurring outside of the perinatal period? A review of the evidence. Focus.

[CR4] Beck CT (2001). Predictors of postpartum depression: Un update. Nursing Research.

[CR5] Besser, A., Vliegen, N., Luyten, P., & Blatt, S. J. (2008). Vulnerability to postpartum commentary on issued raised by Blum (2007). *Psychoanalytic Psychology*, *25*(2), 392–410. 10.1037/0736-9735.25.2.392

[CR6] Bloch M, Meiboom H, Lorberblatt M, Bluvstein I, Aharonov I, Schreiber S (2012). The effect of sertraline add-on to brief dynamic psychotherapy for the treatment of postpartum depression: A randomized, double-blind, placebo-controlled study. The Journal of Clinical Psychiatry.

[CR7] Branquinho M, Rodriguez-Muñoz MF, Rodrigues Maia B, Marques M, Matos M, Osma J, Moreno-Peral P, Conejo-Cerón S, Fonseca A, Vousoura E (2021). Effectiveness of psychological interventions in the treatment of perinatal depression: A systematic review of systematic reviews and meta-analyses. Journal of Affective Disorders.

[CR8] Clarici A, Pellizzoni S, Guaschino S, Alberico S, Bembich S, Giuliani R, Short A, Guarino G, Panksepp J (2015). Intranasal adminstration of oxytocin in postnatal depression: Implications for psychodynamic psychotherapy from a randomized double-blind pilot study. Frontiers in Psychology.

[CR9] Cooper P, Murray L, Wilson A, Romaniuk H (2003). Controlled trial of the short-and long-term effect of psychological treatment of post-partum depression: Impact on maternal mood. The British Journal of Psychiatry.

[CR10] Cox JM, Holden R, Sagovsky R (1987). Detection of postnatal depression. Development of the 10-item Edinburgh postnatal depression scale. The British Journal of Psychiatry.

[CR11] De Crescenzo F, Perelli F, Armando M, Vicari S (2014). Selective serotonin reuptake inhibitors (SSRIs) for post-partum depression (PPD): A systematic review of randomized clinical trials. Journal of Affective Disorders.

[CR13] Driessen E, Hegelmaier LM, Abbass AA, Barber JP, Dekker JJ, Van HL, Jansma EP, Cuijpers P (2015). The efficacy of short-term psychodynamic psychotherapy for depression: A meta-analysis update. Clinical psychology review.

[CR14] First, M. B., Spitzer, R. L., Gibbon, M., & Williams, J. B. (1994). Structured clinical interview for Axis I DSM-IV disorders. 10.1001/archpsyc.1992.01820080032005 1637252

[CR16] Hamilton M (1967). Development of a rating scale for primary depressive illness. British Journal of Social and Clinical Psychology.

[CR17] Jones SH, Thornicroft G, Coffey M, Dunn G (1995). A brief mental health outcome scale; reliability and validity of the Global Assessment of Functioning (GAF). The British Journal of Psychiatry.

[CR18] Kaźmierczak M, Michałek-Kwiecień J, Kiełbratowska B, Karasiewicz K (2020). Parents’ personality and maternal experiences in childcare as predictors of postpartum depression in couples in transition to parenthood. Psychiatria Polska.

[CR19] Kurzweil S (2008). Relational–developmental Therapy Group for postnatal depression. International Journal of Group Psychotherapy.

[CR20] Kurzweil S (2012). Psychodynamic therapy for depression in women with infants and young children. American Journal of Psychotherapy.

[CR21] Leichsenring F, Klein S (2014). Evidence for psychodynamic psychotherapy in specific mental disorders: A systematic review. Psychoanalytic Psychotherapy.

[CR22] Li X, Laplante DP, Paquin V, Lafortune S, Elgbeili G, King S (2022). Effectiveness of cognitive behavioral therapy for perinatal maternal depression, anxiety and stress: A systematic review and meta-analysis of randomized controlled trials. Clinical Psychology Review.

[CR23] Liu X, Wang S, Wang G (2022). Prevalence and risk factors of postpartum depression in women: A systematic review and meta-analysis. Journal of Clinical Nursing.

[CR24] Malhi GS, Bell E, Bassett D, Boyce P, Bryant R, Hazell P, Murray G (2021). The 2020 Royal Australian and New Zealand College of Psychiatrists clinical practice guidelines for mood disorders. Australian & New Zealand Journal of Psychiatry.

[CR25] Miniati M, Callari A, Calugi S, Rucci P, Savino M, Mauri M, Dell’Osso L (2014). Interpersonal psychotherapy for postpartum depression: A systematic review. Archives of Women’s Mental Health.

[CR26] Moayedoddin, A., Moser, D. A., & Nanzer, N. (2013). The impact of brief psychotherapy centred on parenthood on the anxio-depressive symptoms of mothers during the perinatal period. *Swiss Medical Weekly*, *143*(1112), 10.4414/smw.2013.13769. 10.4414/smw.2013.13769 23519551

[CR27] Moher D, Liberati A, Tetzlaff J, Altman DG, Group PRISMA (2009). Preferred reporting items for systematic reviews and meta-analyses: The PRISMA statement. Annals of Internal Medicine.

[CR28] Montgomery SA, Asberg MA (1979). A new depression scale designed to be sensitive to change. British Journal of Psychiatry.

[CR30] Nanzer N, Riguetti-Vetelma M (2009). Use of an easily administered instrument to detect the risk of postpartum depression. Revue Médical Suisse.

[CR31] Nanzer N, Sancho Rossignol A, Righetti-Veltema M, Knauer D, Manzano J, Espasa P (2012). Effects of a brief psychoanalytic intervention for perinatal depression. Archives of Women’s Mental Health.

[CR32] Nillni YI, Mehralizade A, Mayer L, Milanovic S (2018). Treatment of depression, anxiety, and trauma-related disorders during the perinatal period: A ystematic review. Clinical Psychology Review.

[CR34] Shalev, A. Y., Abramowitz, M. Z., & Kaplan-De-Nour, A. (1994). *Structured clinical interview for DSM-IV Patient Edition*. SCID-I/P. Version 2.0; Hebrew Version, 1994).

[CR35] Spitzer RL, Williams JB, Gibbon M, First MB (1992). The structured clinical interview for DSM-III-R (SCID). I: History, rationale, and description. Archives of General Psychiatry.

[CR36] Werner E, Miller M, Osborne LM, Kuzava S, Monk C (2015). Preventing postpartum depression: Review and recommendations. Archives of Women’s Mental Health.

